# B cell phenotypes and maturation states in cows naturally infected with *Mycobacterium avium* subsp. *Paratuberculosis*

**DOI:** 10.1371/journal.pone.0278313

**Published:** 2022-12-07

**Authors:** J. R. Stabel, J. P. Bannantine, S. Humphrey

**Affiliations:** 1 Infectious Bacterial Diseases of Livestock Research Unit, USDA-ARS, National Animal Disease Center, Ames, IA, United States of America; 2 Microscopy Services Department, USDA-ARS, National Animal Disease Center, Ames, IA, United States of America; Cornell University, UNITED STATES

## Abstract

Little is known about the role that B cells play in immune responses to infection with the intracellular pathogen, *Mycobacterium avium* subsp. *paratuberculosis* (MAP). Traditionally, the role of B cells has been constrained to their function as antibody-producing cells, however, antibodies are not thought to play a protective role in mycobacterial infections. The present study was designed to characterize B cell subpopulations as well as activation/maturation states in cattle with paratuberculosis. Peripheral blood mononuclear cells (PBMCs) were isolated from noninfected control cows (n = 8); as well cattle naturally infected with MAP in the subclinical (n = 8) and clinical (n = 7) stage of infection and stimulated with MAP antigen for 6 days. MAP infection resulted in greater numbers of total B cells for clinical cows compared to control noninfected cows. The major subpopulation in freshly isolated PBMCs in clinical cows was B-1a B cells, but this shifted to a composite of both B-1a and B-2 B cells upon stimulation of PBMCs with either MAP antigen or pokeweed mitogen, with higher numbers of B-2 B cells. Early B cells were observed to predominate the population of B cells in PBMCs, with lesser populations of germinal B cells, memory B cells and plasma cells. These subpopulations were elevated in clinical cows upon stimulation of PBMCs with MAP antigen, except for plasma cells which were lower compared to control noninfected cows. Increased numbers of B cells in clinical cows aligned with higher expression of B cell markers such as MAPK1/3, BTG1, Bcl2, CD79A and SWAP70, depending upon in vitro stimulation with either mitogen or antigen. This would indicate that the B cells were capable of activation but were anti-apoptotic in nature. The shift to B-2 B cells in the periphery of clinical cows seems to be indicative of an expansion of memory B cells, rather than plasma cells. This may be a last attempt by the host to control the rampant inflammatory state associated with advanced clinical disease.

## Introduction

Similar to many mycobacterial diseases, paratuberculosis (Johne’s disease) is controlled primarily by T cell responses following infection with *Mycobacterium avium* subsp. *paratuberculosis* (MAP). As an enteric pathogen, MAP is primarily transmitted via fecal-oral route from infected dam to calf, homing to the small intestine of affected animals [1]. After uptake by resident macrophages, MAP can either thwart aspects of macrophage function allowing it to reside and replicate within the host cell or it can result in a cascade of interactive responses with lymphocytes, resulting in clearance from the host. Primary T cell subpopulations, CD4, CD8, and gd TCR T cells, are critical in the control of mycobacterial infections, including MAP, using all tools within their arsenal such as cytokines to activate macrophages as well as cytolytic activity [2, 3]. In contrast, the role that B cells may play in host immune responses to MAP infection has not been extensively studied.

Antibodies secreted by B cells in MAP infection become apparent primarily when infected animals segue from asymptomatic subclinical infection to clinical disease but seemingly provide little benefit to the host in controlling or clearing the infection [4]. Serum antibody is a useful diagnostic tool, particularly on an individual animal basis, as it is a rapid and effective means of detection in a herd. However, the increase in serum antibodies to MAP during progression from subclinical to clinical infection has been associated with a concomitant increase in the numbers of B cells [5–7]. This begs the question as to why the host would respond to unchecked infection by producing higher numbers of B cells, particularly if the B cells are not producing a useful mitigation of infection. Antibodies produced by B cells may provide some benefit via opsonization of the intracellular pathogen, thereby enhancing phagocytosis by the macrophage [8]. It may be more insightful to consider that B cells may play a significant role in maintaining the full complement of immunity necessary to control intracellular infections. This seems likely as neonatal calves as early as 1-month post-challenge with live MAP demonstrated increased percentages of activated B cells (CD25+ and CD45RO+), as well as CD5+ B cells, that didn’t correspond with antibody production [9]. Additional roles for B cells include presentation of antigen to CD4+ T cells with subsequent induction of cytokines that may be part of a key host inflammatory response [8]. Yet there has been a seeming disregard in efforts to define B cell responses or to elucidate B cell subpopulations during MAP infection. However, much of the lag in understanding of B cell function in ruminants, but particularly for cattle, lies in the lack of reagents available to distinguish subpopulations [10]. The latter is an important point as for the most part B cells have been studied as a total population, rather than considered as diverse subpopulations with different phenotypes during disease states [11].

Recent studies have reported that humoral immune responses upon vaccination for mycobacterial pathogens are advantageous for the host, suggesting a protective mechanism [8, 12]. In addition to the functions mentioned above for B cells, non-classical mechanisms may also influence the level of protection. Vaccination with a heat-killed whole-cell vaccine for paratuberculosis resulted in seroconversion of vaccinated calves by 6 months of age, suggesting that B cells are highly capable of responding to antigen at an early age [7]. Serum antibodies coordinated with increased percentages of antigen-specific B cells in vaccinated calves upon stimulation of PBMCs with either johnin purified protein derivative or a whole-cell sonicate of MAP.

One instrument of B cell immunity would be Fc receptor activation of macrophage function downstream, increasing the ability to kill phagocytized mycobacteria. Further, a more substantial non-classical mechanism would be antibody-mediated immunity as a mediator between innate immunity and cell-mediated immunity, both essential components of first line defense against mycobacterial pathogens. This was demonstrated in a study in which serum with antibody titers harvested from patients vaccinated with BCG enhanced mycobactericidal activity of neutrophils and macrophages, while also increasing proliferative responses of CD4 and CD8+ T cells [13]. Further, the presence of B cells has been recently reported in active lesions of lepromatous leprosy, with increasing presence of plasma cells as the major portion of B cell population in lepromatous leprosy [14, 15]. B cells are also heterogenous regarding cytokine production, with secretion of pro-inflammatory cytokines such as IFN-γ and IL-12 primed by Th1 cell interaction with B cells, and secretion of IL-2, IL-13, and IL-4 secretion by B cells primed by Th2 cells. Additionally, regulatory B cells are responsible for IL-10 and TGF-β secretion, much like regulatory T cells. It is clear there exists a complex relationship between B and T cells in the immune response to infectious pathogens.

Transitions in B cell populations, B-1a, B-1b, and B-2, have been demonstrated for cattle, and, particularly for cattle with infectious disease. Markers such as CD5 have allowed the differentiation of B-1 cell subsets, with perhaps the most studied and consistent change during infectious disease being a shift to CD5+ B cells, defined as the B-1a B cell subset. Infections as diverse as *Trypanosoma congolense*, mycobacteria (MAP), and bovine leukosis virus (BLV) have resulted in increased numbers of CD5+ B cells in cattle [16–20]. CD5+ B cells play a key role in T cell-B cell interactions, resulting in antibody secretion to a wide variety of antigens [21]. Additionally, autocrine IL-10 production has been observed for CD5+ B cells upon engagement of the B cell receptor [22], an essential function in tempering local inflammatory responses due to enteric pathogens such as MAP. Further mechanisms of CD5+ B cells need to be discerned as within the CD5+ population, divergent dim and bright subpopulations have been demonstrated in cattle infected with either MAP or BLV [18, 20]. Although a CD5- B cell subpopulation (B-1b) is present as well, it is considered a minor cell population and has less relevance for fighting infection. B-2 B cells are synonymous with more conventional B cells, i.e., antibody secreting B cells and their function has been studied more extensively, with documented activation and differentiation pathways, leading to terminally differentiated memory cells or plasma cells [23].

The present study was designed to characterize changes in the number of circulating B cells, as well as shifts in B cell subpopulations, and B cell maturation states due to a progression of Johne’s disease from asymptomatic subclinical disease to a clinical disease state. These data provide critical insights as to whether differences in the subpopulations of B cells are responsible for the dysregulation of host immunity during MAP infection, leading to advanced clinical disease.

## Materials and methods

### Animals

Holstein dairy cows ranged in age from 4 to 9 years in this experiment and were placed in three groups consisting of 8 non-infected healthy cows, 8 cows naturally infected with MAP but asymptomatic (i.e., subclinical), and 7 cows with the clinical form of Johne’s disease. Infection was monitored bacteriologically for the fecal shedding of MAP using standard culture methods on Herrold’s egg yolk agar medium containing mycobactin J, amphotericin, nalidixic acid, and vancomycin (Becton Dickinson, Sparks, MD) as previously described [24]. Serologic tests were used to further characterize status of infected animals. Serum was harvested from whole blood and assayed for the presence of MAP antibodies by commercial ELISA (Herdchek, IDEXX, Westbrook, ME) and bovine IFN-γ was measured in plasma using the Bovigam test kit (Prionics, La Vista, NE) as described by the manufacturer. Animals categorized as clinical had ELISA antibody titers averaging 1.13 S/P ratio and fecal shedding averaged 27541 cfu of MAP/g of feces. Cows in the subclinical treatment group were ELISA-negative and averaged less than 230 cfu of MAP/g of feces. Infected animals in both subclinical and clinical stage of infection had positive antigen-specific IFN-γ results (Abs_450nm_MPS-Abs_450nm_NS = 0.44±0.21 and 0.25±0.11, respectively). Overall, subclinical cows demonstrated positive antigen-specific IFN-γ responses and negligible to low serum antibody titers and shed MAP intermittently and at low levels in their feces. Cows were categorized as clinical if they were shedding MAP in their feces, had positive serum antibody titers and moderate to low antigen-specific IFN-γ. Subclinical cows were asymptomatic, whereas clinical cows demonstrated signs such as loose stool to watery diarrhea, weight loss, and submandibular edema. All animals were housed in American Association for Accreditation of Laboratory Animal Care-accredited facilities and all animal related procedures were approved by the IACUC (National Animal Disease Center, Ames, Iowa). Infected cows were housed separately on-site from healthy control cows to prevent cross-contamination between groups.

### Cell culture

Briefly, peripheral blood mononuclear cells (PBMCs) were isolated from the buffy coat fractions of blood. PBMCs were resuspended in RPMI-1640 medium (Gibco, Grand Island, NY) supplemented with 10% (v/v) fetal bovine serum (FBS; Atlanta Biologics, Atlanta, GA), penicillin-streptomycin (100×, 10,000 U/ml each; Gibco); MEM non-essential amino acids solution (100×, Gibco); MEM amino acids solution (50×, Gibco); 2 mM L-glutamine (Gibco); 1 mM sodium pyruvate (100×, Gibco); and 50 μM 2-mercaptoethanol (Gibco) at 4.0 x 10^6^/mL in 24-well flat-bottomed plates (Corning Incorporated, Corning, NY) at 39°C in 5% CO_2_ in a humidified atmosphere for 24 hr. Duplicate wells were set up for each animal for the following in vitro treatments: medium only (nonstimulated, NS), pokeweed mitogen (PWM, Sigma; 10 μg/ml), and a whole-cell sonicate preparation of MAP (MPS; strain 167; NADC; 10 μg/ml). Plates were then removed from the incubator, centrifuged at 1500 rpm for 10 min, and cell-free supernatants were harvested, and stored at -20°C until cytokine measurement was performed. For RNA harvesting, cells were lifted off the plate with ice-cold PBS, transferred to microfuge tubes, and centrifuged at 5600 rpm for 5 min to pellet cells. After decantation of PBS, RNA cell protect solution was added to each tube and cells were resuspended. Cells were then stored frozen at -80°C until RNA was processed for cDNA and gene expression analyses were performed. An additional set of 48-well plates was incubated for either 3 days (NS, PWM) or 6 days (NS, MPS) and cells were harvested for flow cytometric analyses. A series of 4 experiments was conducted, with each study expanding the scope of experimentation. All data from the experiments are comprehensively summarized within the study but focused on the final experiments as more reagents became available for use. Data from prior experiments were reproduced within the final experiment.

### Flow cytometric analysis

**Fig 1:**
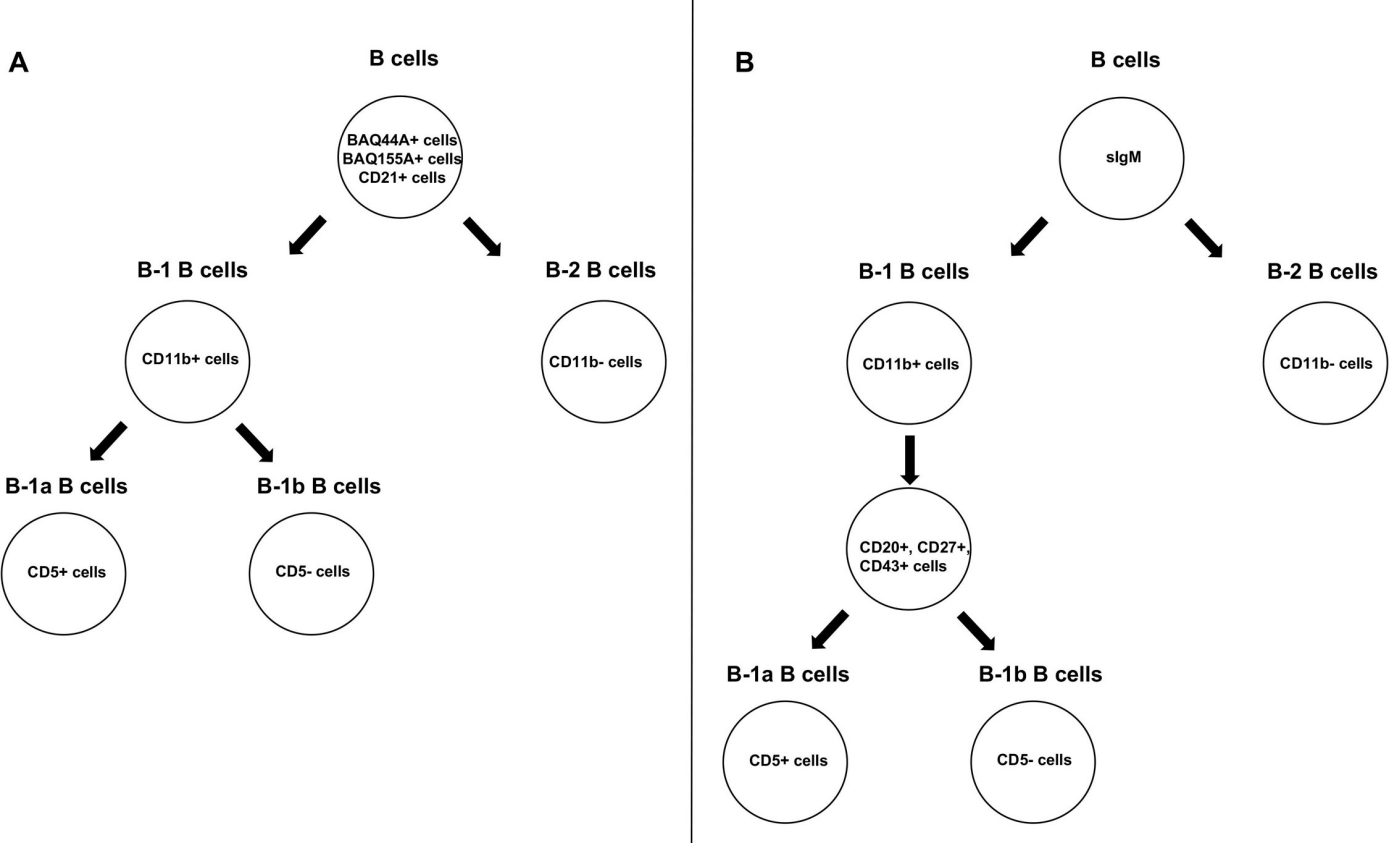
Diagram demonstrating the partitioning of B cells by flow cytometric analyses of PBMCs isolated from blood of noninfected control cows and cows naturally infected with *M*. *avium* subsp. *paratuberculosis*. B cells were partitioned into B-1a, B-1b and B-2 subpopulations using BAQ44A, BAQ155A, and CD21 as primary B cell markers, followed by CD11b, and CD5 as subset markers (A), and using sIgM as primary B cell marker, followed by CD11b, CD20, CD27, CD43, and CD5 as subset markers (B).

Combinations of different primary antibodies were used to phenotype the B cells present in freshly isolated PBMCs, as well as PBMCs cultured with PWM, and MPS. The first experiments were designed to identify B cells at different maturation stages using key B cell markers for cattle. Total B cells were partitioned within the freshly isolated and cultured PBMCs using the primary monoclonal antibody to bovine sIgM (Fig 1A). Within that B cell population, subpopulations were determined using directly conjugated primary antibodies for B-B2 (BAQ44A), B-B4 (BAQ155A), and CD21 (GB25A) B cell markers (Fig 1A and Table 1). To further differentiate the B cell subpopulations into B-1a, B-1b, and B-2 cells, primary antibodies to CD5 and CD11b were employed as shown in the flowchart (Fig 1A and Table 1). However, upon further reading of the literature we chose to refine the separation of subpopulations, characterizing the total B cell population by sIgM staining, followed by primary antibodies to CD20, CD27, and CD43 to identify the B-1 subpopulation more specifically within the CD11b+ population, followed by CD5 to further separate out B-1a and B-1b populations (Fig 1B and Table 1). Additional staining was conducted to discern B cell maturation subpopulations across infection groups. A comprehensive series of antibodies to cell surface markers (Table 1) was utilized to parse B cells into different subsets based upon maturity. Subpopulations included total B cells, germinal B cells (Pre-B), early B cells, memory B cells, and plasma B cells. Primary antibodies used to categorize these subpopulations against markers sIgM, CD21, CD27, CD28, CD40, CD45R, CD80, CD86, CD138, and MHCII are shown in Table 1. The bovine CD138 antibody was a kind gift from Drs. Kelcey Dinkle and Lindsay Fry, USDA-ARS, Pullman, WA. All antibodies were combined in a cocktail prior to addition to cells. The staining protocol for cells was conducted by gently resuspending the freshly isolated or cultured cells and transferring 50 μl of cell suspension to wells of 96-well round bottom plates (Corning Incorporated, Corning, NY) Live/Dead cell staining with eFluor 780 fixable dye was performed as per manufacturers’ instructions (ThermoFisher, cat. # 65-0865-14). Cells were then resuspended in 50 μl of Cell Staining Buffer (BioLegend cat. # 420201) containing the primary antibody cocktail (Table 1) and incubated at RT for 15 min. The cells were centrifuged at 500g at RT for 3 min. and the supernatant discarded. Next, the cells were resuspended in 50 μl of Cell Staining Buffer containing the secondary antibody cocktail (Table 1) and incubated at RT for 15 min. The cells were then centrifuged at 500g at RT for 3 min. and the supernatant discarded. At this point the cells were washed twice with 200 μl of PBS and then resuspended in 50 μl of Cell Staining Buffer containing the directly conjugated antibody cocktail (Table 1) and incubated at RT for 15 min. The cells were centrifuged at 500g at RT for 3 min., the supernatant discarded and washed with 200 μl of PBS and then resuspend in 200 μl of Stabilizing Fixative solution (BD Bioscience, cat. # 338036) and let sit at RT for 10 min. A final centrifugation at 500g at RT for 3 min. was performed, the supernatant discarded, and the cells resuspended in PBS for data collection on the cytometer (BD Bioscience, BD FACSymphony A5). Analysis of the data was done using FlowJo software (BD Bioscience). Gating was performed by plotting SSC-A versus FSC-A, followed by live cells, followed by doublet discrimination as shown in representative histogram flow charts in S1 and S2 Figs. A Boolean tool was used to group the subpopulations by desired markers (and, or, not directives) as demonstrated in S1 Dataset.

**Table 1:** Primary and secondary antibodies used to discriminate B cell subpopulations.

Cell marker	Direct Ab	Primary Ab	Source	Product #	Secondary Ab
CD5^1^^-^^3^		B29A-IgG_2a_	WSU	BOV2013	PE
					Biolegend#407108
CD11b^1^^-^^3^	CC126-FITC		Bio-Rad	MCA1425F	
CD20	MEM-97- AF647		Novus	NBP1-44634AF647	
CD21^3^		BAQ15A-IgM	WSU	BOV2032	AF647
					Biolegend#406526
CD21^3^	LT21-PerCP		Biorbyt	Orb623596	
CD27^1^^-^^3^^,^ ^6^^-^^7^	M-T271-PE		BD Biosciences	562297	
CD28^5^	CC219-PE		Bio-Rad	MCA5779PE	
CD40^4^^-^^7^		ILA158A-IgG_1_	WSU	BOV2107	BV711
					BD Biosciences#565786
CD43^1^^-^^3^		CO.44B8-IgG_1_	Bio-Rad	MCA1096GA	BV711
					BD Biosciences#565786
CD45R^2^	IVA103-AF350		Novus	NB400-492AF350	
CD80^6^	IL-A159-FITC		Bio-Rad	MCA2436F	
CD86^6^		IL-A190-IgG_1_	Bio-Rad	MCA2437GA	AF647
					Biolegend#406618
CD138^7^		IgM	USDA-ARS	N/A	BV421
					Biolegend#406532
MHCII^4^^-^^7^		TH16A-IgG_2a_	WSU	BOV2115	BUV805
					BD Biosciences#749396
sIgM^1^^-^^7^		Pig45A2-IgG_2b_	WSU	BOV2060	BUV395
					BD Biosciences#743180

^1^Markers used to distinguish B-1a B cells: sIgM+, CD5+, CD11b+, CD27+, CD43+.

^2^Markers used to distinguish B-1b B cells: sIgM+, CD5-, CD11b+, CD27+, CD43+

^3^Markers used to distinguish B-2 B cells: sIgM+, CD5-, CD11b-, CD21+, CD27-, CD43-.

^4^Markers used to distinguish Early B cell subpopulation: sIgM+, CD40+, MHCII+.

^5^Markers used to distinguish Activated Germinal B cell subpopulation: sIgM+, CD28+, CD40+, CD45R+, MHCII+.

^6^Markers used to distinguish Memory B cell subpopulation: sIgM+, CD27+, CD40+, CD80+, CD86+, MHCII+, CD45R-.

^7^Markers used to distinguish Plasma B cell subpopulation: sIgM+, CD27+, CD138+, CD4

### Cytokine analyses of cell culture supernatants

Bovine IFN-γ, IL-10, IP-10, and TNF-α were determined in cell culture supernatants using the Milliplex Bovine Cytokine/Chemokine multiplex assay (Millipore Sigma, St. Louis, MO). The assay, based upon a Luminex xMAP format with magnetic bead technology, was conducted using a MAGPIX analyzer (ThermoFisher Scientific) according to manufacturer’s instructions. Concentrations (pg/ml) of each cytokine were quantified using Milliplex Analyst Sofware (Millipore Sigma) by referencing to a standard curve for each cytokine. IL-7 was determined separately using a standard curve comprising serial dilutions of bovine IL-7 standard (31.25–2000 pg/ml; PBP006; Serotec) for quantification.

### RNA extraction and reverse transcription

RNA was extracted from MDM after incubation with live MAP or PBMCs after 24 hr of culture with in vitro treatments (NS, MPS) as described above. Briefly, cells from duplicate wells were harvested after centrifugation of plates at 1500 rpm for 5 min and lysed with 350 μl of buffer RLT (Qiagen, Valencia, CA). RNA was isolated using an RNeasy Mini Kit (Qiagen) according to the manufacturer’s directions and eluted from the column with 40 μl of RNase-free water (Ambion, Austin, TX). Total RNA (500 ng) was reverse transcribed using SuperScript III (Invitrogen, Carlsbad, CA) with 150 ng of random hexamers, 10 mM dNTPs and 40 units of RNaseOut (Invitrogen), according to the manufacturer’s directions. Samples were heated to 65°C for 5 min and then reverse transcribed at 50°C for 60 min. The resulting cDNA were stored at −80°C until used in real-time PCR.

### Cytokine gene expression

Real-time PCR was performed using custom Taqman Gene Expression Assays for bovine IFN-γ, IL-4, IL-7, IL-10, BCL2, BTG1, MAPK1, MAPK3, SWAP70, and CD79a (Life Technologies, Grand Island, NY) according to the manufacturer’s directions for relative quantitation. Target gene assay ID numbers are presented in Table 2. Briefly, 4 μl of cDNA template was added to a 20 μl reaction mixture containing Taqman Universal PCR Master Mix and Gene Expression Assay working stock consisting of forward and reverse primers and FAM-MGB probe. Eukaryotic 18S rRNA endogenous control (FAM-MGB probe, non-prime limited; Invitrogen) was used as an internal control to normalize RNA content between samples. The NS sample was used as the calibrator. All reactions were performed in triplicate, and data were analyzed with the 2^−ΔΔCt^ method.

**Table 2:** Real-time PCR bovine cytokine gene expression^1^.

Cytokine	Assay ID#
IL-4	Bt03211898_m1
IL-10	Bt03212725_m1
IL-7	Bt03210251_m1
IFN-γ	Bt03212722_g1
Bcl2	Bt04259486_m1
BTG1	Bt03249590_m1
MAPK1	Bt03216714_m1
MAPK3	Bt03276141_m1
SWAP70	Bt03269802_m1
CD79a	Bt03259267_m1

^1^Taqman Gene Expression Assays (Life Technologies, Grand Island, NY)

### Statistics

Data were analyzed using the PROC Mixed procedure of the Statistical Analysis System (SAS Institute, Cary, NC). Values were reported as least square means ± the standard error of the mean. When significant effects (*P* < 0.05) due to infection or *in vitro* treatment were observed, a means comparison was conducted using the Tukey-Kramer post hoc test.

## Results

**Fig 2:**
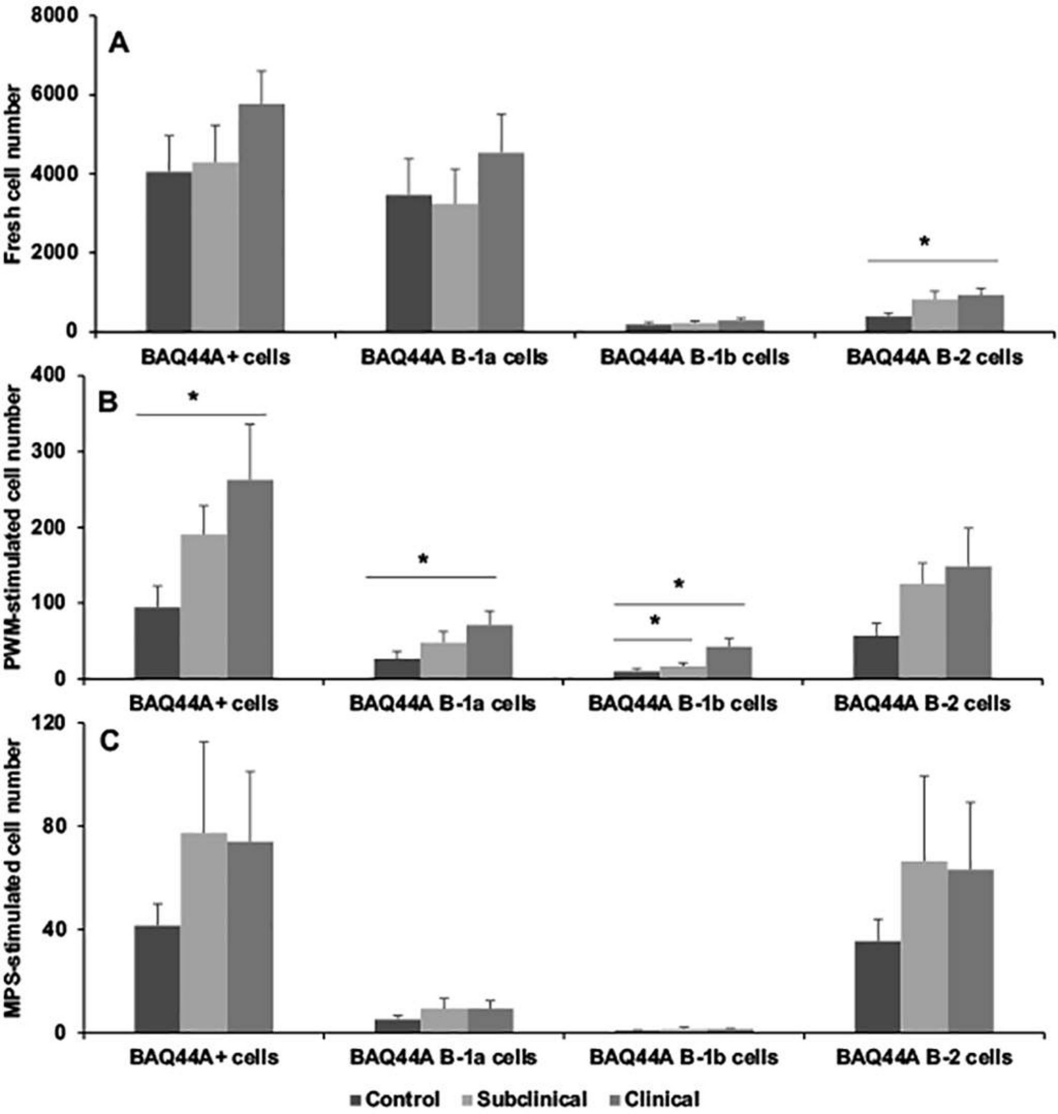
Flow cytometric staining of PBMCs isolated from blood of noninfected control cows and cows naturally infected with *M*. *avium* subsp. *paratuberculosis* using BAQ44A antibody as the primary B cell marker. B cells were further partitioned using CD11b and CD5 antibodies for B cell subpopulations within PBMCs that were freshly isolated (A); incubated with pokeweed mitogen (B; 10 μg/ml); or with a whole cell sonicate (MPS) of *M*. *avium* subsp. *paratuberculosis* (C; 10 μg/ml) for 6 days in vitro.

**Fig 3:**
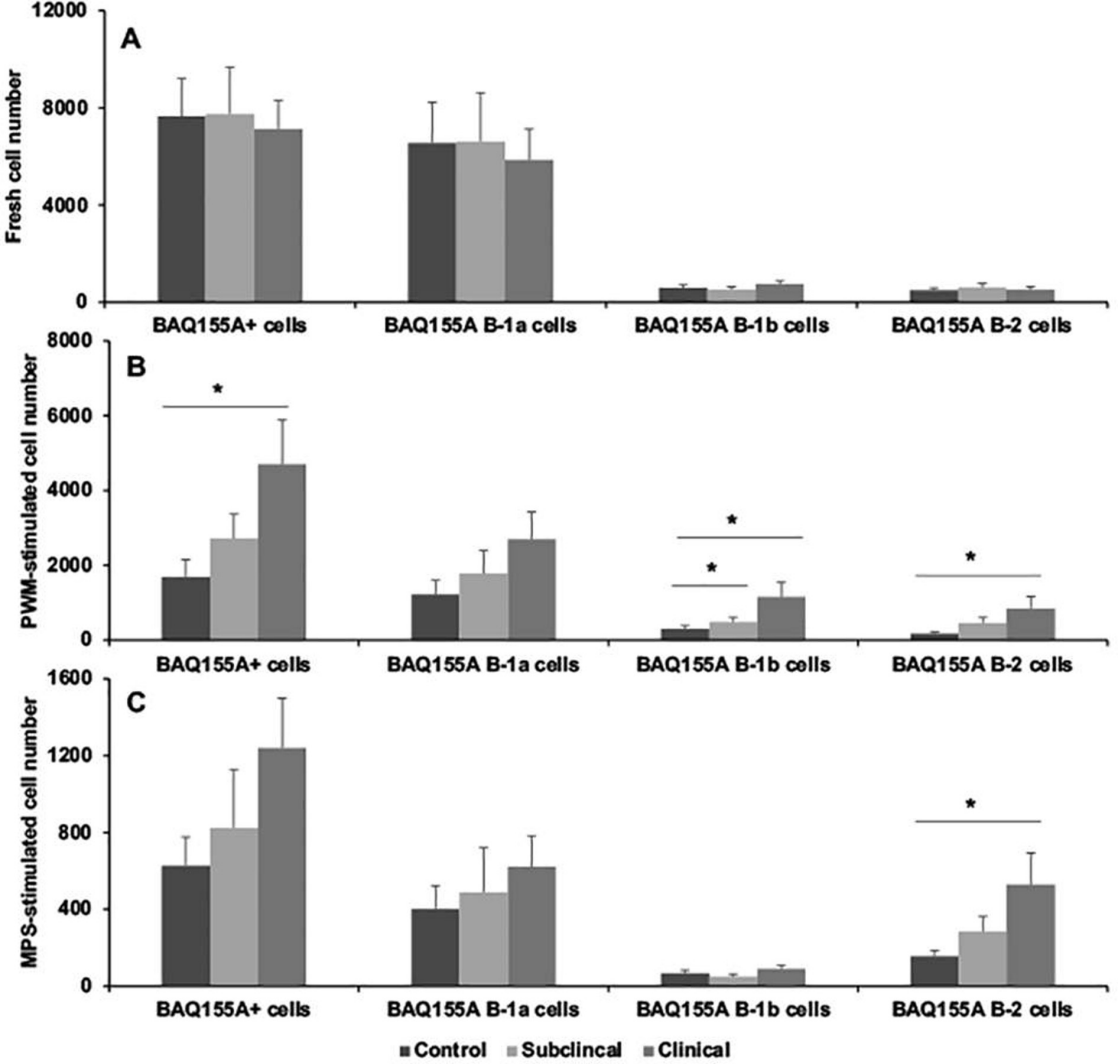
Flow cytometric staining of PBMCs isolated from blood of noninfected control cows and cows naturally infected with *M*. *avium* subsp. *paratuberculosis* using BAQ155A antibody as the primary B cell marker. B cells were further partitioned using CD11b and CD5 antibodies for B cell subpopulations within PBMCs that were freshly isolated (A); incubated with pokeweed mitogen (B; 10 μg/ml) for 3 days; or with a whole cell sonicate (MPS) of *M*. *avium* subsp. *paratuberculosis* (C; 10 μg/ml) for 6 days in vitro.

**Fig 4:**
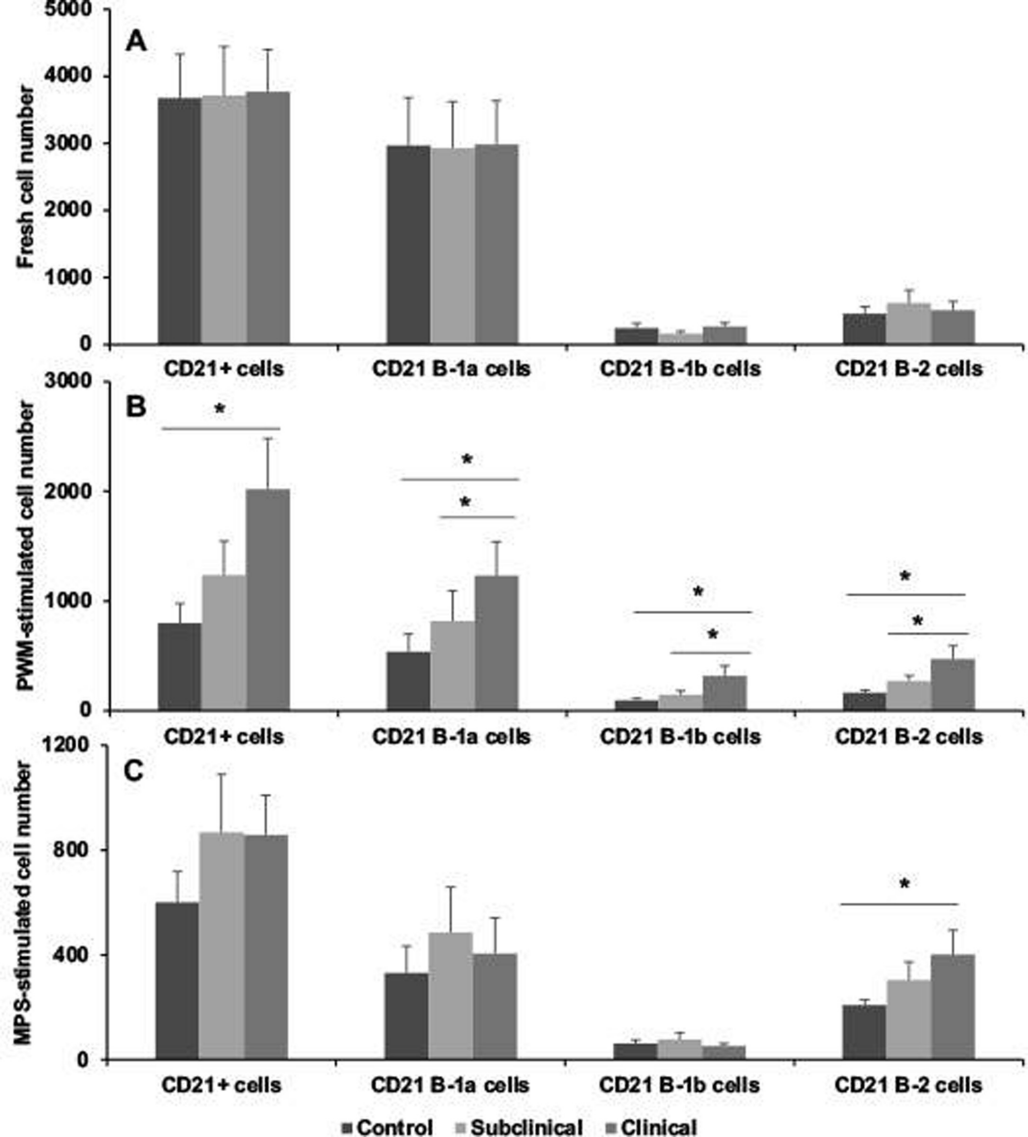
Flow cytometric staining of PBMCs isolated from blood of noninfected control cows and cows naturally infected with *M*. *avium* subsp. *paratuberculosis* using CD21 antibody as the primary B cell marker. B cells were further partitioned using CD11b and CD5 antibodies for B cell subpopulations within PBMCs that were freshly isolated (A); or incubated with pokeweed mitogen (B; 10 μg/ml) for 3 days; or with a whole cell sonicate (MPS; 10 μg/ml) of *M*. *avium* subsp. *paratuberculosis* (C; 10 μg/ml) for 6 days in vitro.

**Fig 5:**
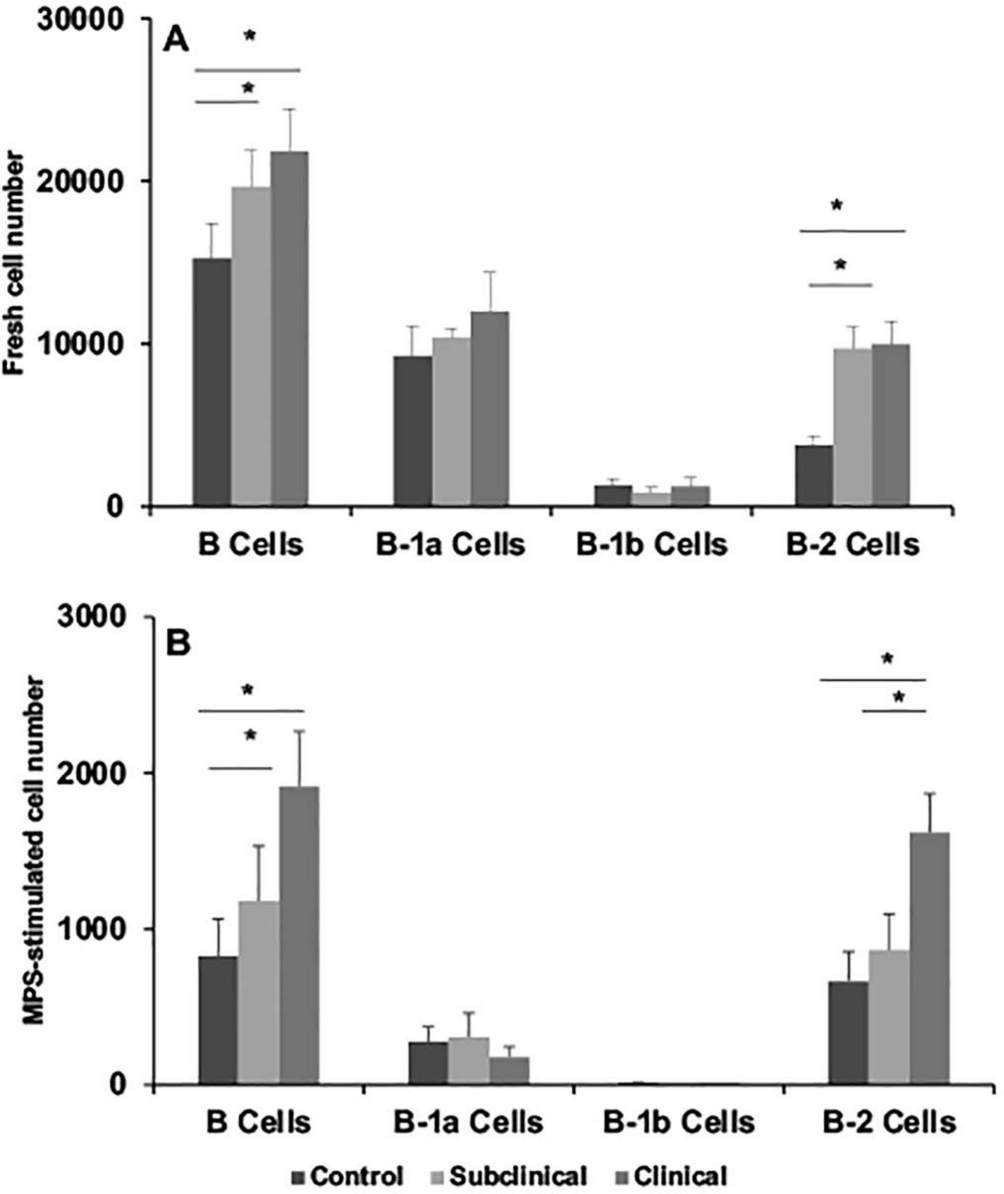
Flow cytometric staining of PBMCs isolated from blood of noninfected control cows and cows naturally infected with *M*. *avium* subsp. *paratuberculosis* using sIgM antibody as the primary B cell marker. B cells were further partitioned using CD11b, CD20, CD27, CD43, and CD5 antibodies for B cell subpopulations within PBMCs that were freshly isolated (A); or incubated with a whole cell sonicate (MPS; 10 μg/ml) of *M*. *avium* subsp. *paratuberculosis* (B) for 6 days in vitro.

The effects of infection status on the partitioning of the B cell population are presented in Figs 2–5. Results for BAQ44A+ B cells demonstrated a trend towards greater number of total B cells in naturally infected cows in the clinical stage of disease both in freshly isolated PBMCs and in cultured cells stimulated with MPS antigen, with a significant (*P* < 0.05) expansion noted in PWM-stimulated cells (Fig 2). Separation of the B cell population into B-1a, B-1b, and B-2 subsets resulted in significantly (*P* < 0.05) higher B-2 B cells for clinical cows compared to control noninfected cows in the cells freshly isolated from the cow (Fig 2A). Stimulation of PBMCs with PWM for 3 days further defined the expansion (*P* < 0.05) of B-1a and B-1b cells in clinical cows, along with a strong trend for higher B-2 cells for both subclinical and clinical cows (Fig 2B). Although stimulation of cells with MPS also invoked similar trends, the results were nonsignificant due to increased variability within the treatment groups. Cells defined by the BAQ155A marker demonstrated similar patterns for the PWM-stimulated PBMCs, with higher total BAQ155A+ B cells, B-1b cells and B-2 cells noted for clinical cows (Fig 3B). Additionally, B-2 cells were also higher (*P* < 0.05) for clinical cows after stimulation of PBMCs with MPS (Fig 3C). No significant differences were observed in freshly isolated cells due to infection status. This lack of effect for freshly isolated cells was true for cells stained for CD21 marker as well (Fig 4A). Significant (*P* < 0.01) trends for higher numbers of CD21+ cells, along with the key subset populations, aligned with the results noted for the BAQ44A and BAQ155A markers for clinical cows (Fig 4). In the final experiments we used a more comprehensive total B cell marker (sIgM) and, therefore, repeated the same measurements to examine the output (Fig 5). In freshly isolated cells, both total B cells and B-2 cells demonstrated significant (*P* < 0.05) increases for infected cows compared to noninfected controls. The pattern observed in cells stimulated with the MPS antigen for 6 days showed a significant (*P* < 0.05) upregulation of total B cells and B-2 cells only for clinical cows (Fig 5B).

**Fig 6:**
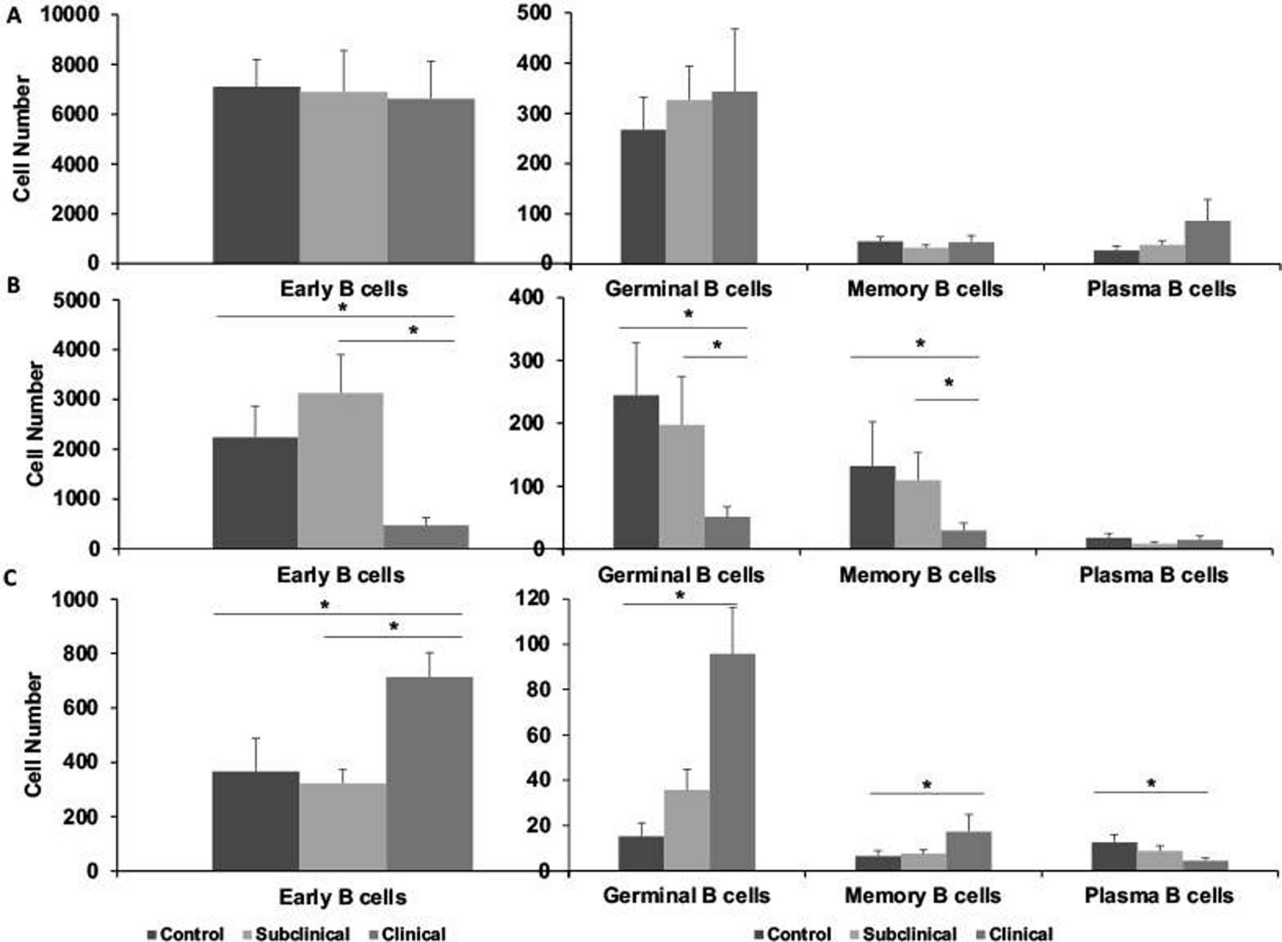
Flow cytometric staining of PBMCs isolated from blood of noninfected control cows and cows naturally infected with *M*. *avium* subsp. *paratuberculosis* to separate B cells by stage of maturity. Partitioning of B cells into maturation states of early B cells, germinal B cells, memory B cells, and plasma cells was performed using combinations of antibodies referenced in Table 1. PBMCs were isolated (A); stimulated with pokeweed mitogen (B; 10 μg/ml) for 3 days; or with a whole cell sonicate (MPS) of *M*. *avium* subsp. *paratuberculosis* (C; 10 μg/ml) for 6 days in vitro.

To further refine the categorization of B cells by maturity status, we stained freshly isolated PBMCs, along with cells cultured with PWM and MPS, with combinations of monoclonal antibodies to specific cell markers as defined in Table 1. There were no significant differences of note within freshly isolated PBMCs due to infection status of the cows (Fig 6A). However, a trend (*P* < 0.08) was observed in higher numbers of plasma B cells in the clinical cows. Upon stimulation of PBMCs with PWM for 3 days a downregulation (*P* < 0.01) of different subpopulations was noted for clinical cows compared to subclinical cows and noninfected control cows (Fig 6B). In contrast, stimulation with MAP antigen (MPS) shifted towards an expansion (*P* < 0.05) of total B cells, early B cells, memory B cells, and germinal B cells (Figs 6C and S3). The exception to this was a significant (*P* < 0.01) decrease in plasma B cells noted for clinical cows.

**Fig 7:**
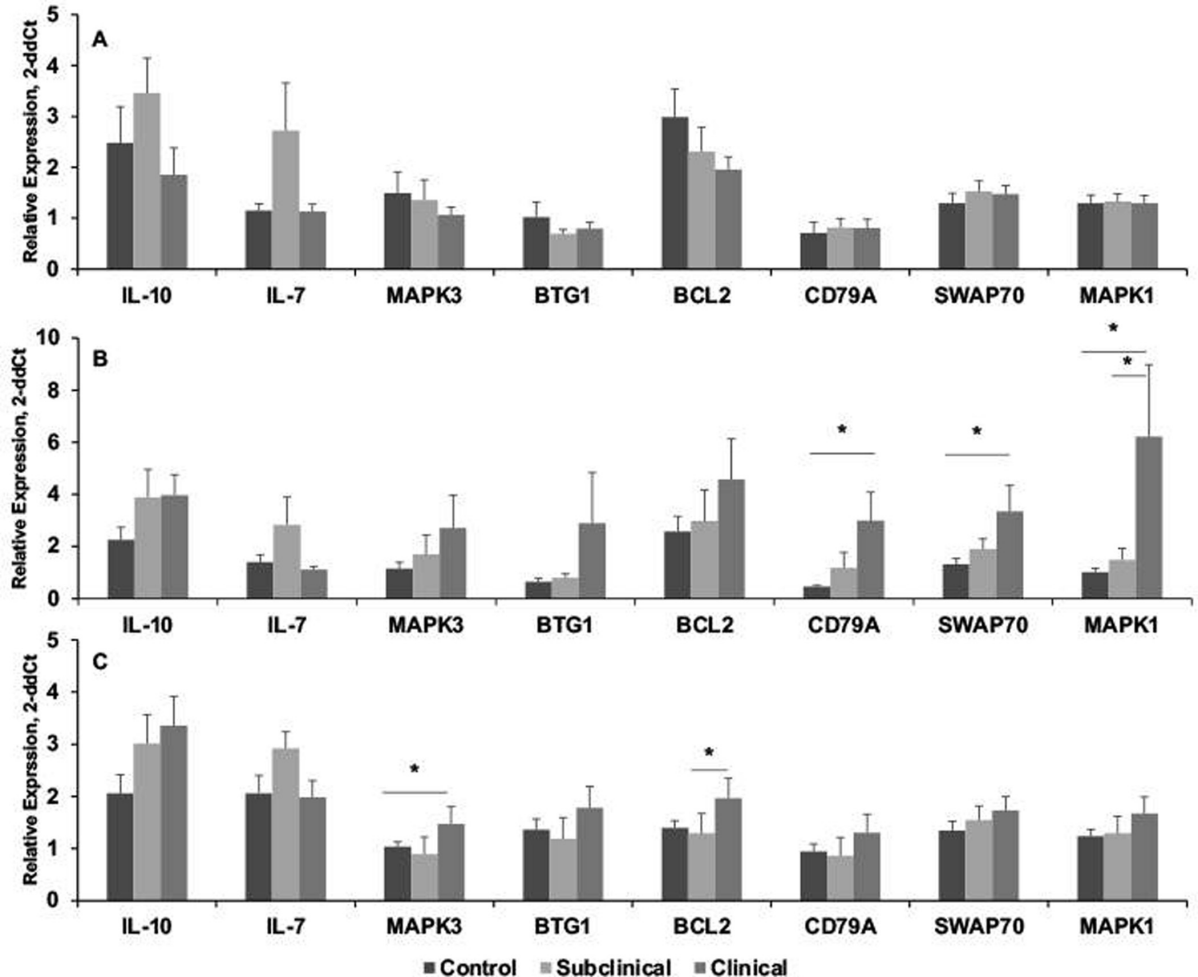
Relative gene expression (2^-ddCt^) for B cell markers and cytokines by PBMCs isolated from control noninfected cows and cows naturally infected with *M*. *avium* subsp. *paratuberculosis*. PBMCs were stimulated for 24 h in vitro with a whole cell sonicate (MPS; 10 μg/ml) of *M*. *avium* subsp. *paratuberculosis*, RNA was extracted, and gene expression analyses were performed using Taqman Gene Expression Assays (Life Technologies, Grand Island, NY).

Endeavoring to understand the interplay between cell types as a potential explanation for the expansion of B cells observed in clinical cows, cytokine secretion and expression was measured in cultured PBMCs. There were trends in higher amounts of secreted IFN-g, IP-10, and IL-17 in infected cows, however, these did not seem to correlate with a switch in B cell subpopulations (S4 Fig). A trend in higher IL-10 for clinical cows was observed concomitant with a decrease in IL-4 (*P* < 0.06) for infected cows (S4 Fig). Additional measurement of secreted IL-7 yielded no differences due to infection status. Potentiation of IL-10 expression for infected cows after stimulation of PBMCs with either PWM or MPS further aligned with this theory (Fig 7). Interestingly, IL-7 expression trended higher for subclinical cows regardless of in vitro treatment of PBMCs. Expression of IL-4 yielded no conclusive results as PWM stimulation potentiated higher levels of IL-4 for infected cows but upon stimulation of cells with MPS, IL-4 expression was barely measurable. This occurred in several experiments with different sets of cows, with a decrease in average relative gene expression of IL-4 from 500 for PWM-stimulated cells to an average of 2 for MPS-stimulated cells, regardless of infection status of cows. In addition, specific markers for B cell subpopulations and activation were measured on PBMCs in freshly isolated cells, as well as upon stimulation with PWM or MPS. No significant differences were noted in freshly isolated cells due to infection status of cows. Upon stimulation of cells with PWM, expression of MAPK3, BTG1, BCL2 trended higher, whereas CD79a, SWAP70, and MAPK1 expression were significantly (*P* < 0.01) higher for clinical cows compared to control cows or subclinical cows (Fig 7B). Upon culture of PBMCs with MPS, MAPK3 and BCL2 were significantly (*P* < 0.01) increased for clinical cows (Fig 7C).

## Discussion

A hallmark characteristic of advanced clinical disease for cattle infected with MAP is a shift towards higher numbers of B cells in the peripheral blood [25]. It is unclear why this shift occurs and if it is reflective of a breakdown in the host immune system, allowing for progression of the disease state, or if it is a penultimate effort by the host to fight back against the intracellular pathogen. Previously, our laboratory demonstrated that cattle with clinical disease had higher percentages of total B cells (70%) within the mononuclear cell population compared to control noninfected and subclinical cows (40%) [5]. Perhaps even more interesting was the finding that the B cells isolated from clinical cows were refractory to MAP antigen but did proliferate with concanavalin A stimulation. In contrast, B cells from subclinical cows responded well to both antigen and mitogen stimulation [5]. Further, shifts in subpopulations of CD5+ B cells were demonstrated in infected cows, compared to noninfected controls [26]. The CD5 marker is a surface glycoprotein expressed on both early and mature T cells but is also expressed on B cells [27]. Its role on the surface of B cells has not been adequately defined but has been suggested to act as an activation marker and/or adhesion molecule. Interestingly, CD5 can effectively delineate between B-1 and B-2 B cells, and even further demarcate B-1a cells (CD5+) from B-1b (CD5-) cells [27], and this applies in cattle as well [10]. Cattle infected with bovine leukosis virus have been the most extensively studied, with observed increases in the CD5+ B cell population for infected cattle, depending upon the association with persistent lymphocytosis [17, 20, 28]. Additionally, some earlier work in cattle infected with trypanosomes revealed increased CD5+ B cells [29]. Our previous work showed that CD5+ B cells were lower for cows infected with MAP, with concomitant increases in CD5- B cells [26]. The CD5+ B-1a cells in the naturally infected cows shifted from a CD5+^dim^ population to an increase in CD5+^bright^ B cells, suggesting that this transition may be reflective of a regulatory mechanism during progression of infection. More recently, dairy cows infected with BLV demonstrated a shift in CD5 expression on CD5+ B cells with an increased subpopulation of CD5+^dim^ B cells upon stimulation of PBMCs with keyhole limpet hemocyanin [20]. Although no known function for these CD5+ subpopulations has been elucidated yet, the reproducibility in more than one cattle study suggests potential functional differences during infectious disease. There is much speculation as to specific roles for CD5 on B cells, including modulation of the B cell receptor (BCR) with effects on downstream signaling pathways [30]. These signaling pathways include key pathways for cellular survival, metabolism, and cytokine production, all factors that would affect efficient responses to an infectious pathogen [30]. CD5+ B cells also are considered an innate-like B cell population that can act as antigen-presenting cells, rapidly producing antibodies in response to infection [31]. Collectively, these roles suggest that CD5+ B cells would be advantageous to the host in response to infection. These studies lead us to explore further the characteristics of B cells and their phenotypes in naturally infected cows in different stages of disease.

Results from the present study clearly demonstrate that MAP infection skews the total B cell population, regardless of which antibody was used to quantitate B cells (BAQ44A, BAQ155A, CD21, or sIgM). The original intent was to determine if the 3 markers in Figs 2–4 would differentiate B cell populations in the host, by either activation state or state of maturation. These B cell markers are woefully uncharacterized in cattle, but some preliminary information was available to suggest that they might be useful to delineate the ontogeny of B cells. Previously, these antibodies were used to demarcate the B-B2 (BAQ44A) and B-B4 (BAQ155A) epitopes on B cells in cattle [32, 33]. The B-B2 marker is present on most B cells (sIgM) with equivalence to the C19+ marker, but one disadvantage is that it also labels T cells [34]. The presence of the B4 marker is an indicator of B cell activation and can be present on normal and mitogen-activated B cells as well as B cell tumors, with predominant labeling of the B-1 population [32]. In contrast, the CD21 marker is found most frequently on mature B cells and is lost on plasma cells [35]. The lack of antibody reagents for cattle has hampered the ability to differentiate B cell subsets, but progress is being made (24), Most recently, an 8-color panel of antibodies was developed to distinguish B cell phenotypes in cattle [36]. This panel of antibodies to B cell markers such as CD20, CD21, CD40, CD71, and CD138 were arrayed to allow characterization of naïve B cells, regulatory B cells, memory B cells, plasmablasts, and plasma cells. The array aligned somewhat with the immunophenotyping conducted in the present study, but we utilized a Boolean tool to group the subpopulations by desired markers (and, or, not directives), allowing for further distinction.

Despite the distinctions between the 3 major B cell markers, the pattern of staining and delineation between B-1 and B-2 B cells due to infection status of cows was similar regardless of which antibody was used. Clinical cows consistently had higher numbers of B cells in either PWM mitogen- or MPS-stimulated PBMC cultures, whereas the number of B cells for subclinical cows aligned somewhere in-between control noninfected cows and clinical cows, suggesting there is a temporal progression of B cell numbers that parallels disease state. The key subpopulation that increased in MAP infection was the B-2 subpopulation and this was the most significant and reproducible in response to MAP antigen stimulation of cells. This was true as well for PWM-stimulated cells but using a polyclonal stimulant also engendered expansion of B-1a and B-1b subpopulations for clinical cows, depending upon the B cell marker used. The increases in B-2 cells aligns with the appearance of serum antibodies to MAP as cows progress to clinical disease. In the group of cows utilized within the present study serum antibodies to MAP as measured by commercial ELISA averaged 0.13 and 1.13 S/P for subclinical and clinical cows, with S/P values above 0.6 considered positive. The increases in B-1a and more consistently in B-1b cells after stimulation of cells with PWM contrasted to the effects of antigen stimulation. Overall, the responses to antigen versus mitogen in clinically affected cows is interesting, suggesting that clinical cows may exploit the ability to mount B cell responses aligning with both innate and adaptive immunity [37]. B-1 B cells are the major subpopulation that seem to provide protection against bacterial pathogens in the event of an infection, with the ability to self-renew and demonstrate long-term surveillance in the host [38]. The shift from B-1 B cells to B-2 B cells in cows with MAP infection, particularly upon exposure to MAP antigen, may be indicative of a key point in the progression from subclinical to clinical disease.

B cell subsets were segregated into maturation states as optimally as possible with available bovine reagents and/or cross-reactive antibodies from other species, some markers can be shared between cell types, likely resulting in some overlap. The highest percentage of circulating B cells are naïve whereas the remaining third of the total population is memory B cells, aligning itself with results shown in the present study. We demonstrated that clinical cows had higher numbers of each subset of B cells upon stimulation of the PBMCs with MAP antigen compared to either noninfected controls or subclinical cows. The exception for that trend was the decrease in number of plasma cells observed for clinical cows after exposure to antigen. Plasma cells are generally short-lived in cell culture systems, lasting about 3–5 days and can begin apoptosis within 1 day of culture. It is likely that our extended culture system reflects a loss in the number of plasma cells over time as these are terminally differentiated cells, with the additional caveat that circulating numbers of plasma cells are relatively low [39]. Additionally, it has been demonstrated that antigen exposure will determine the lifespan of plasma cells with corresponding decay rates between antigen and antibody production [40]. This is an interesting finding and the decrease in plasma cells would seem at odds with the noted increases in B-2 cells for clinically affected animals. However, although both B-2 and plasma cells secrete antibodies they are not necessarily synonymous with one another and differ in function. B-2 cells are more classical B cells that function as APC, produce cytokines such as IL-2, IL-4, TNF-a, and IL-6, and secrete antibodies [41], whereas plasma cells are terminally differentiated and secrete clonal antibodies [39]. Whether the antibodies secreted by B-2 cells and plasma cells differ functionally is unknown. It is also of note that increased numbers of plasma cells in the intestine and associated mesenteric lymph nodes in both cattle and sheep have been demonstrated in MAP infection, resulting in a more localized function for this B cell phenotype [42, 43]. This may account for the low number of plasma cells in the peripheral blood noted for infected cows in the present study. A lesser population that was influenced by clinical status of cows were the germinal B cells, a population characterized in the periphery as a transitional cell between the naïve cell population and memory/plasma cell differentiation [44, 45]. This may be a critical point in the event of advanced clinical disease at the germinal center if the proper phenotypes for protective immunity are not expanded. If the B cell population is being diverted away from the development of plasma cells, this would lessen the availability of antibodies to interact with MAP via opsonization, resulting in unchecked inflammatory responses [46]. Another interesting observation was the divergent response to PWM as the cell stimulant, resulting in decreased cell populations for clinical cows across all phenotypes and maturation states. An exception was the similar plasma cell number invoked by polyclonal expansion with PWM, regardless of infection status, further suggesting that antigen recall responses shifted the population in the germinal center to a memory-type phenotype.

Upregulated gene expression of basic markers of B cell activation and proliferation aligned with the expansion of B cell subpopulations for clinical cows in the present study. Very little information is available in the literature on regulation of these genes in cattle, particularly in a disease state, therefore, we extrapolated many of their functions from studies conducted with other species. CD79a and SWAP70 are key genes involved in the B cell receptor complex, with roles in B cell activation [47, 48]. CD79a is associated with early maturation of B cells and is generally not present on plasma cells [49], corresponding with the lower numbers of plasma cells we observed for clinical cows. Increased CD79a expression was also observed in B cell lymphomas in cattle with BLV as compared to healthy cattle [50]. Further, MAPK1/3 genes play key roles in B cell activation with impacts on T cell immunity, including induction of T cell anergy (MAPK3/ERK2) [51, 52]. This is a key point as T cell anergy has been observed in cases of severe clinical disease in cattle and sheep with paratuberculosis [6, 53], and is not associated with Treg cell induction [54]. Interestingly, BTG1 and Bcl2 are both anti-proliferation genes, resulting in increased survival of terminally differentiated cells [55]. If B cells in cows with advanced clinical disease are thwarted from typical apoptotic pathways, then accumulation of greater numbers of B cells in the periphery would be expected. In addition to its role as a promoter of B cell activation and antibody production IL-7 is noted for multiple functions encompassing both innate and adaptive host immunity [56]. The higher level of IL-7 expression observed for subclinical cows is interesting as one critical role for IL-7 is the activation and survival of peripheral T cells [56, 57]. It is conceivable that IL-7 mediates a B-T cell interaction that maintains control of infection in subclinical cows. The increased expression of IL-10 for infected cows, regardless of status, is reflective of the presence of B and T regulatory cells within MNC population, both subpopulations that effectively temper immunopathology in the host [58, 59].

Finally, it was of further interest that B cell numbers noted for cows with subclinical paratuberculosis in the present study fell somewhere in-between those for control noninfected cows and cows with clinical disease. Yet association of activation/proliferation markers on PBMCs from subclinical cows aligned more often with control noninfected cows, even after exposure to MAP antigen. This suggests again that B cells play a role in progression of advanced disease and perhaps provide a balance within the host [60]. One such role may be the modulation of the T cell environment, with roles for B cells and secreted antibodies in acquired immunity to infectious pathogens [8]. Antibodies can regulate CD4+ T cells in acute mycobacterial infection by formation of Ag-Ab complexes. An observed elevation of antibodies occurring in advanced tuberculosis may be coordinate with increased IL-10 [60], much as we see with clinical paratuberculosis. The regulatory effects of IL-10 on IFN-γ, an essential component of controlling mycobacterial infections, have been characterized to dampen the effects of excessive inflammatory responses in the host but can also retard critical T cell immunity that is required by the host to control intracellular pathogens. Antigen presentation by B-1 B cells can induce expansion of Th1/Th17 T cells, in contrast to Treg cell proliferation induced by B-2 B cells [61]. This aligns with previous publication by our laboratory demonstrating that clinical disease resulted in a shift to Treg-mediated immunity in cattle naturally infected with MAP [62].

In conclusion, the progression of disease state from a subclinical asymptomatic state to a more advanced clinical disease state suggests that B cell immunity is transitory in the cow and responds to changes in T cell-mediated immunity by evolving to different B cell phenotypes. This seems apparent with the shift to an increased B-2 B cell population in cows with clinical disease, a subpopulation that functions in a regulatory manner, inducing IL-10 production, and tempering inflammatory responses in the host. Infection of cattle with MAP resulted in increased CD5+ B cells, particularly the B-1a B cell subpopulation, aligning with results noted for other infectious pathogens in cattle. Most notable for clinical cows was the increase in number of early B cells, germinal B cells, and memory B cells, with a concomitant decrease in plasma B cells upon stimulation of PBMCs with MAP antigen. This agrees with the increased expression of CD79a observed for clinical cows. The increased expression of anti-apoptotic markers in cows with clinical disease helps explain the preponderance of B cells in the periphery and further suggests that a regulatory phenotype may be necessary in advanced disease. The shift in B cell number and phenotype that is observed in chronic mycobacterial infections, with long-term progression from a controlled asymptomatic disease state to a more advanced clinical state, may be indicative of a final effort by the host to control infection.

## Supporting information

S1 Fig Representative flow cytometry histogram demonstrating the flow of analyses for partitioning of B cells for one cow. PBMCs isolated from cows were harvested and incubated with cocktails of primary antibodies for specific targets. B cells were partitioned into subpopulations using the Boolean gating tool.
(TIF)

S2 Fig Representative flow cytometry histogram demonstrating the flow of analyses for partitioning of B cells for one cow. PBMCs isolated from cows were harvested and incubated with cocktails of primary antibodies for specific targets. B cells were partitioned into subpopulations using the Boolean gating tool.
(TIF)

S3 Fig A representative flow cytometry histogram demonstrating B cell maturation states for PBMCs isolated from blood of noninfected control cows and cows naturally infected with *M*. *avium* subsp. *paratuberculosis*. The histogram depicts partitioning of cells into total live mononuclear cell population (A), total B cells gated within live cells (sIgM; B), and early B cells gated within live B cells (C) for one control noninfected cows (Cow 1507), one subclinical cow (Cow 1459) and one clinical cow (Cow 8339).
(TIF)

S4 Fig Cytokine secretion from PBMCs. Results of secreted cytokines, IFN-γ, IL-4, IL-17A, IL-10, IP-10, TNF-α (pg/ml) from PBMCs isolated from blood of noninfected control cows and cows naturally infected with *M*. *avium* subsp. *paratuberculosis* (Subclinical and Clinical).
(TIF)

S1 File Representative flow cytometry Boolean gating tool for panel of markers for partitioning of B cells into subpopulations for one cow. Diagram delineates stepwise protocol of gating on entire mononuclear cell populations, followed by live cell discrimination, followed by single cell discrimination, and then a cocktail of antibodies to B cell markers within that discrete population.
(TIFF)

S1 Dataset (XLSX)
