# Supplementary figures and images for: B cell phenotypes and maturation states in cows naturally infected with Mycobacterium avium subsp. Paratuberculosis
Source: PLoS One. 2022 Dec 7;17(12):e0278313. doi: 10.1371/journal.pone.0278313 (PMC9728927; doi:10.1371/journal.pone.0278313)

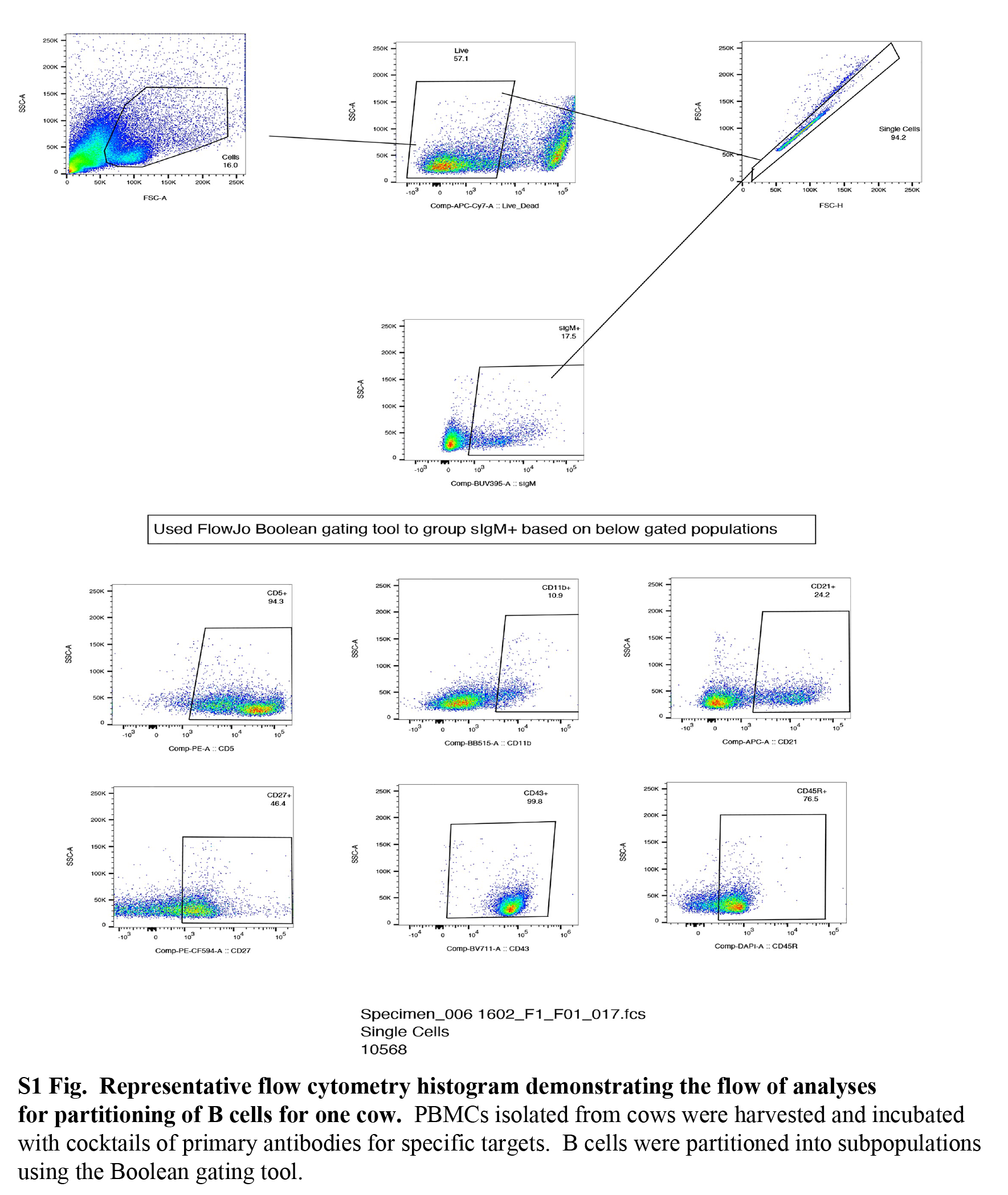

Supplement: S1 Fig — PBMCs isolated from cows were harvested and incubated with cocktails of primary antibodies for specific targets. B cells were partitioned into subpopulations using the Boolean gating tool. (TIF) [file pone.0278313.s001.tif]

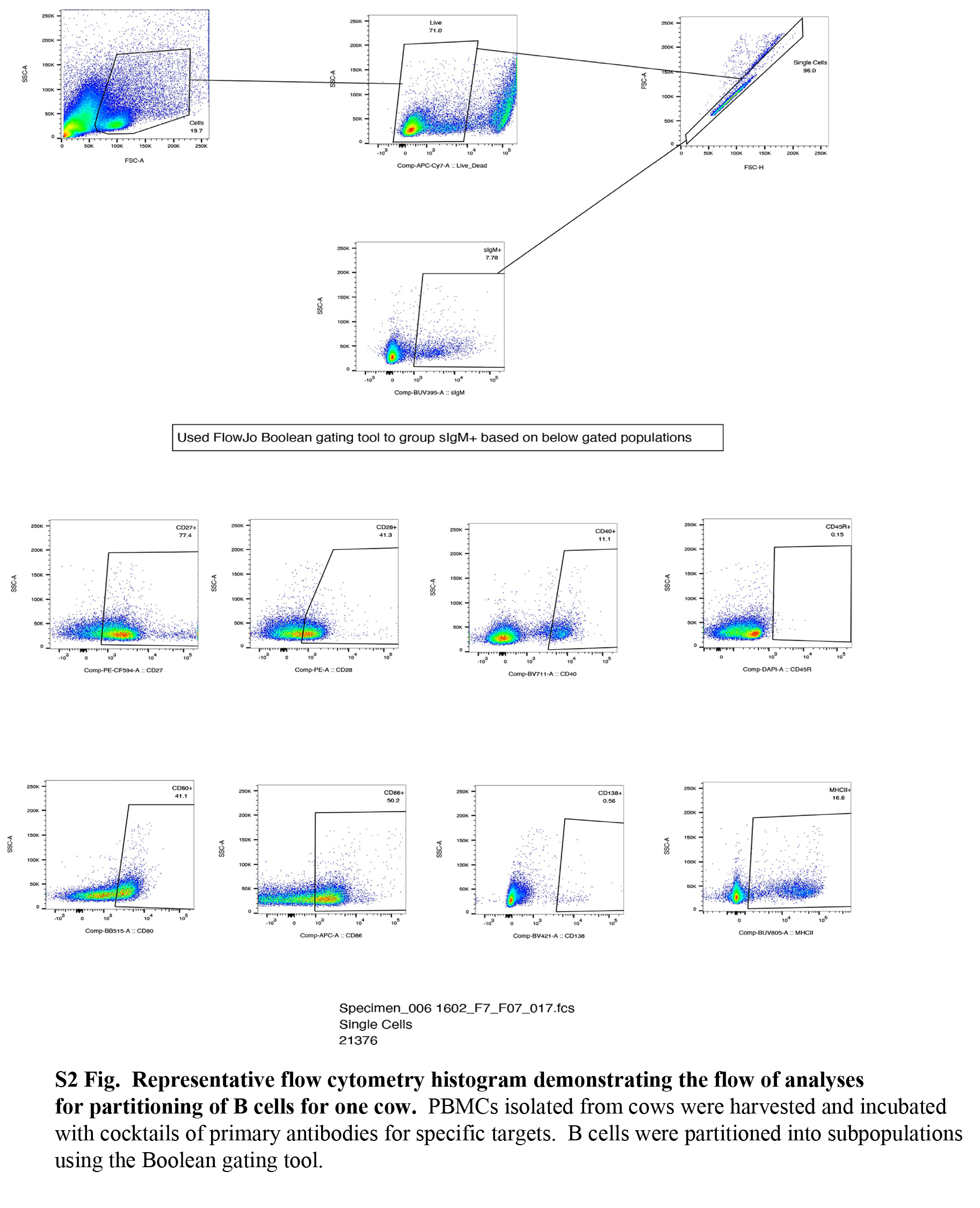

Supplement: S2 Fig — PBMCs isolated from cows were harvested and incubated with cocktails of primary antibodies for specific targets. B cells were partitioned into subpopulations using the Boolean gating tool. (TIF) [file pone.0278313.s002.tif]

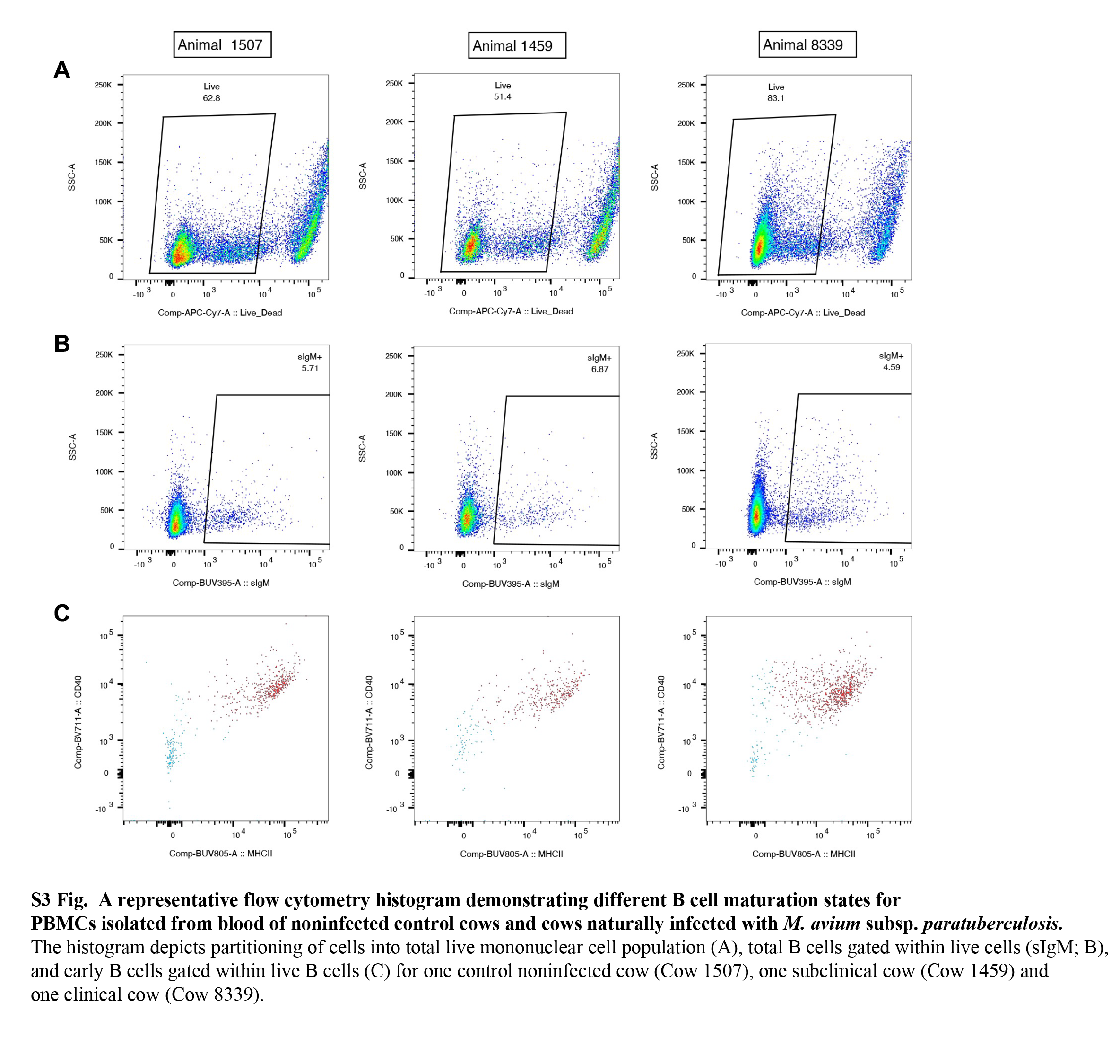

Supplement: S3 Fig — The histogram depicts partitioning of cells into total live mononuclear cell population (A), total B cells gated within live cells (sIgM; B), and early B cells gated within live B cells (C) for one control noninfected cows (Cow 1507), one subclinical cow (Cow 1459) and one clinical cow (Cow 8339). (TIF) [file pone.0278313.s003.tif]

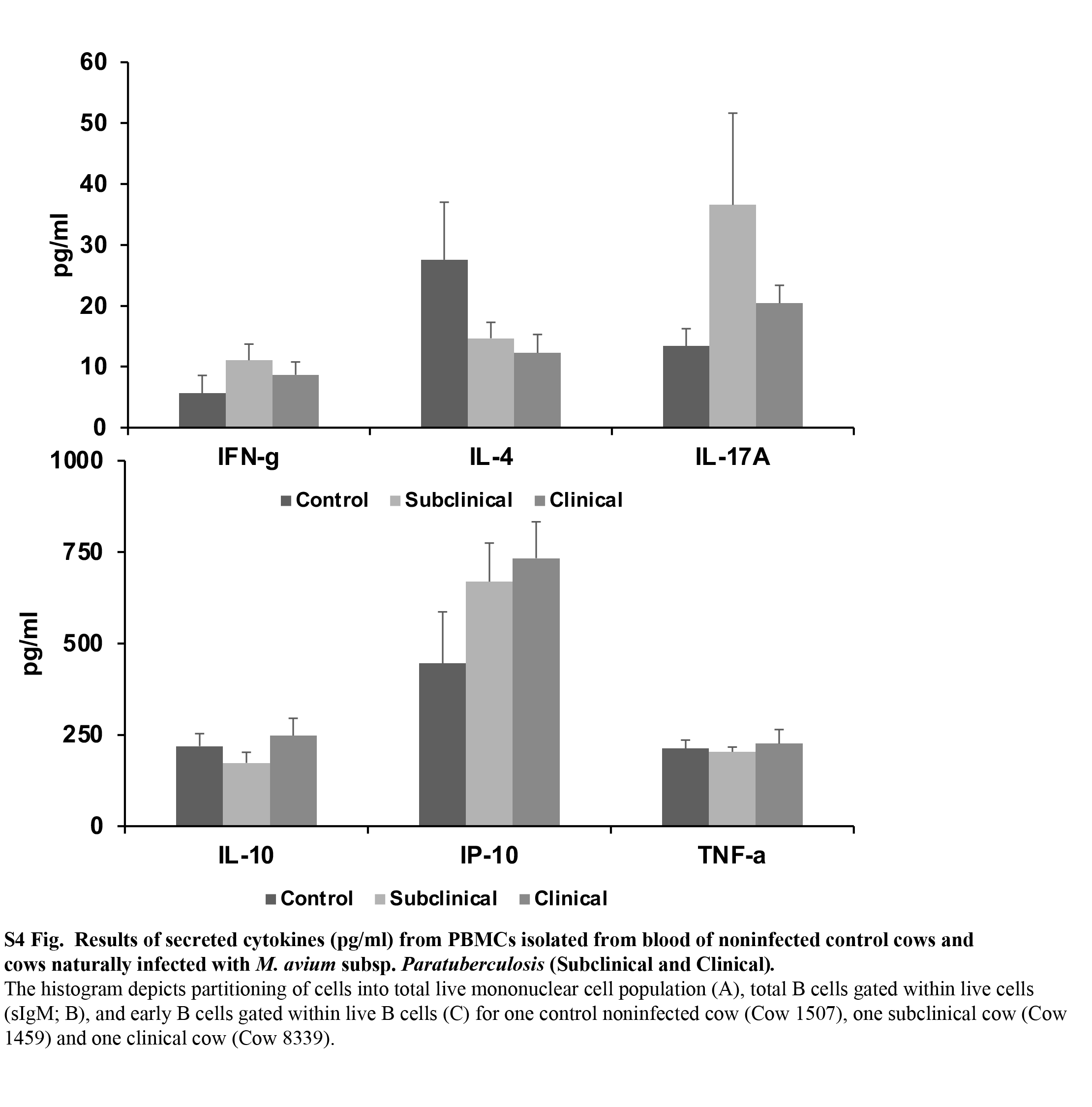

Supplement: S4 Fig — Results of secreted cytokines, IFN-γ, IL-4, IL-17A, IL-10, IP-10, TNF-α (pg/ml) from PBMCs isolated from blood of noninfected control cows and cows naturally infected with M. avium subsp. paratuberculosis (Subclinical and Clinical). (TIF) [file pone.0278313.s004.tif]

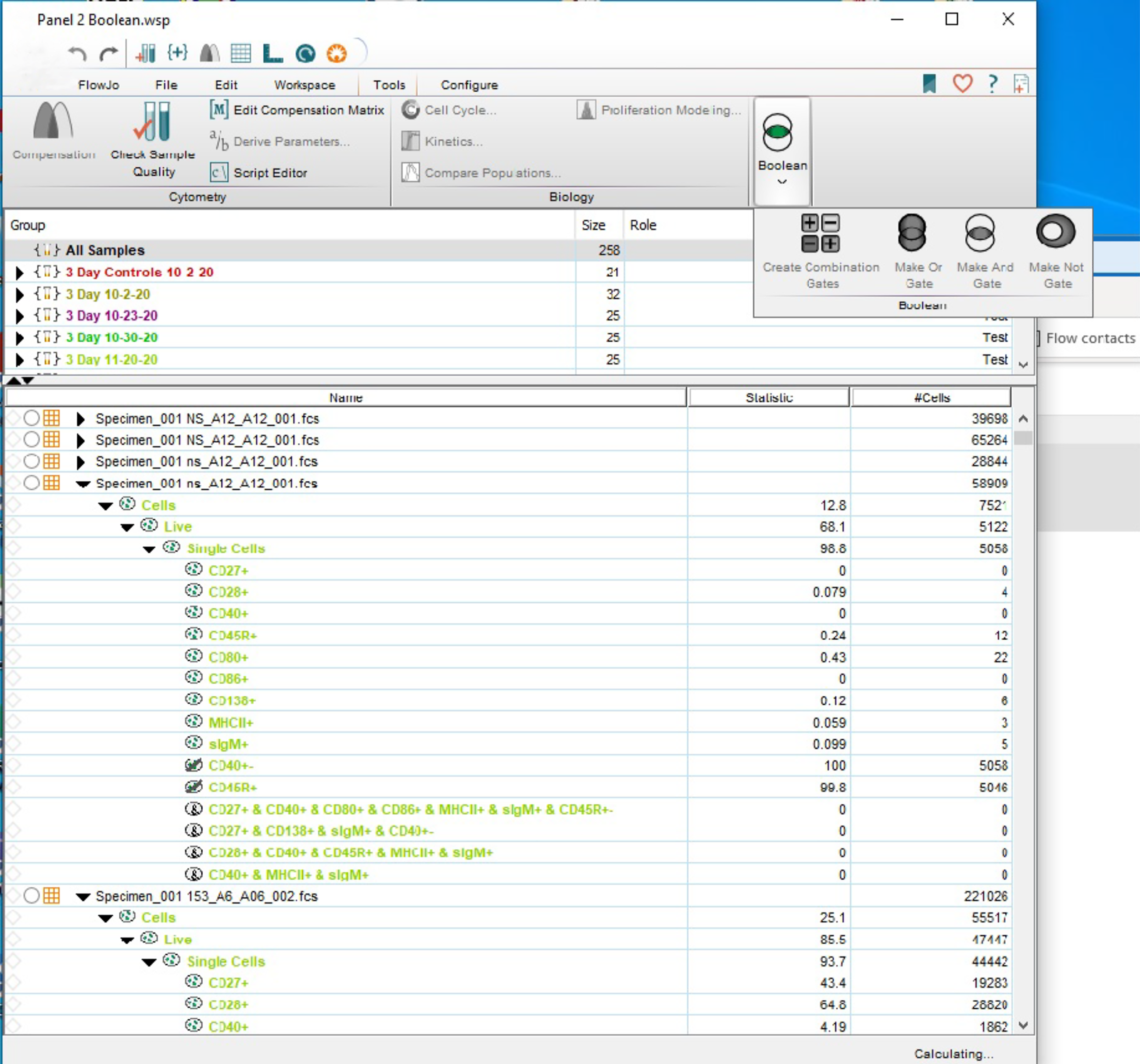

Supplement: S1 File — Diagram delineates stepwise protocol of gating on entire mononuclear cell populations, followed by live cell discrimination, followed by single cell discrimination, and then a cocktail of antibodies to B cell markers within that discrete population. (TIFF) [file pone.0278313.s005.tiff]
